# ﻿Nomenclatural and taxonomic notes on *Rubusdavidianus* Kuntze and *R.viburnifolius* Franch

**DOI:** 10.3897/phytokeys.211.85777

**Published:** 2022-10-11

**Authors:** Tiran Huang, Liping Yu, Juntao Li, Wenhe Wang, Aizhen Yang, Wenping Wang, Cong Wang, Mingfeng Yang, Hong Wang, Lanqing Ma

**Affiliations:** 1 Beijing Advanced Innovation Center for Tree Breeding by Molecular Design, Beijing University of Agriculture, Beijing 102206, China Beijing University of Agriculture Beijing China; 2 Key Laboratory for Northern Urban Agriculture of Ministry of Agriculture and Rural Affairs, Beijing University of Agriculture, Beijing 102206, China University of Chinese Academy of Sciences Beijing China; 3 College of Life Sciences, University of Chinese Academy of Sciences, Beijing 100049, China Beijing University of Agriculture Beijing China; 4 College of Landscape Architecture, Beijing University of Agriculture, Beijing 102206, China University of Chinese Academy of Sciences Beijing China

**Keywords:** new synonyms, *Rubusdavidianus*, *R.lambertianus*, *R.malifolius*, *R.viburnifolius*, species identity

## Abstract

Critical examinations of specimens, with literature reviews, have shown that *Rubusdavidianus* is conspecific with *R.lambertianus*. Therefore, we treat *R.davidianus* as a new synonym within *Rubus*. We propose a new name, *Rubusloirensis* Ti R. Huang nom. nov. to replace the later homonym of *R.pycnanthus* Genev. Additionally, lectotypification of three names, *R.davidianus* Kuntze, *R.malifolius* Focke and *R.viburnifolius* Franch., are designated here after examination of previous works.

## ﻿Introduction

*Rubus* L. is one of the most complicated taxonomic groups in the plant kingdom and is distributed worldwide from the lowland tropics to the subarctic region (Thompson 1995). Intraspecific/interspecific morphology and ploidy variability, apomictic tendencies and the capability of many species to hybridise widely across multiple ploidy levels, complicate *Rubus* taxonomy (Bammi and Olmo 1966; Alice et al. 2001; Mimura et al. 2014; Carter et al. 2019). In response to this, taxonomists disagree broadly about the number of species in the genus, with different estimates ranging from 250 (Mabberley 2017), 700 (Robertson 1974; Lu and Boufford 2003), 750 (Lu 1985), 600–800 (Thompson 1995) to more than 1000 (Jennings 1988). The most recent global taxonomic treatment of this genus was conducted by Focke in 1910, 1911 and 1914 and 12 subgenera were defined. Phylogenetic results over the past 25 years suggest that Focke’s subdivisions of *Rubus* are not monophyletic and large-scale taxonomic revisions are necessary. While working on the infrageneric re-classification of *Rubus*, we found that the taxonomic status of *R.davidianus* Kuntze and *R.viburnifolius* Franch. should be verified, especially in China (Lu and Boufford 2003). The names related to these two species, *Batidaeaviburnifolia* Greene, *R.pycnanthus* Genev. and *R.viburnifolius* Focke, were also checked.

**Figure 1.**
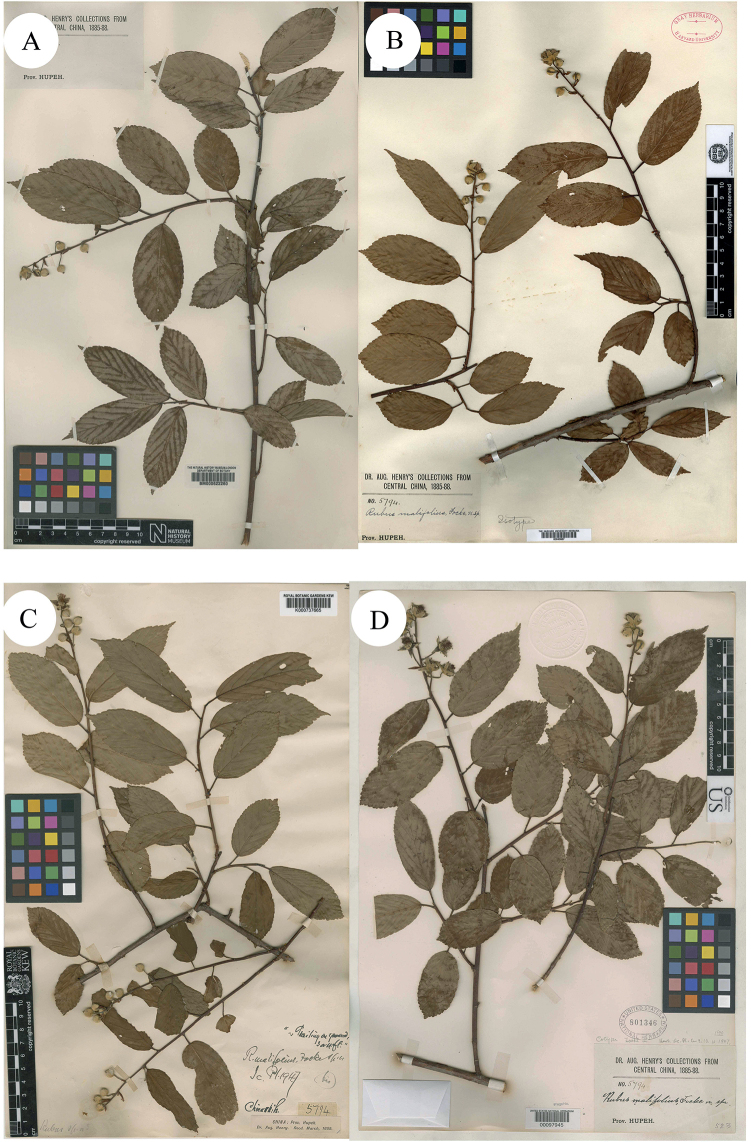
**A–D** Syntypes of *R.malifolius* Focke. Specimen barcodes: BM000622260, GH00040667, K000737665 and US00097945, respectively.

*Rubusmalifolius*Focke (1890) was published, based on the collection from Chienshih, Hubei, China, A. Henry, 1885, no. 5794 (Syntypes BM000622260!; GH00040667!; K000737665!; US00097945!; Fig. 1A–D). Its critical characteristics were described as “Shrubs scandent. Leaves simple, elliptic or oblong-elliptic, base subrounded, margin inconspicuously shallowly serrate, apex acuminate, rarely acute, abaxial surface of leaves tomentose. Inflorescences terminal, racemes, bracts caducous, linear-oblong, pubescent initially, apex acute to shortly acuminate. Calyx abaxially densely tomentose-villous; sepals ovate to triangular-ovate or lanceolate. Petals white or white with pink spots, round, both surfaces thinly pubescent, base shortly clawed. Stamens many, slightly villous, anthers hirtose. Pistils much longer than stamens, ovary glabrous, styles glabrous, apex clavate. Aggregate fruit purplish-black at maturity, compressed globose, glabrous”.

**Figure 2.**
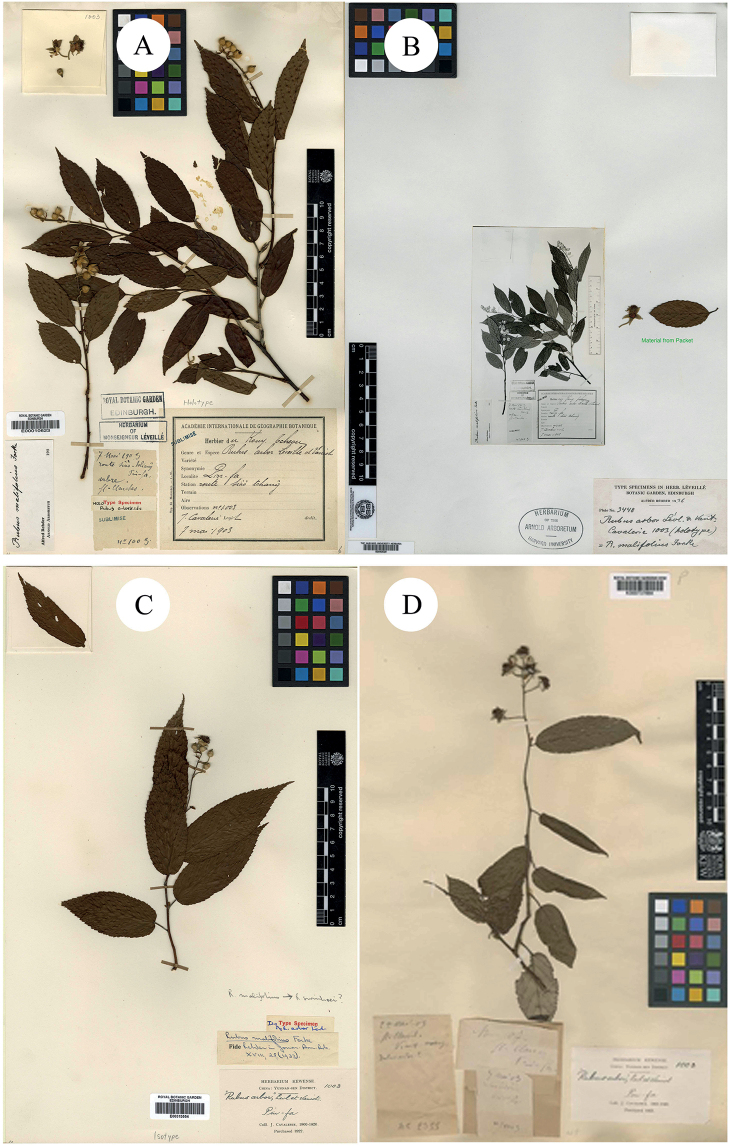
**A** Holotype of *R.arbor* H. Lév. & Vaniot **B–D** Isotypes of *R.arbor* H. Lév. & Vaniot. Specimen barcodes: E00010623, A00040529, E00313554 and K000737664, respectively.

**Figure 3.**
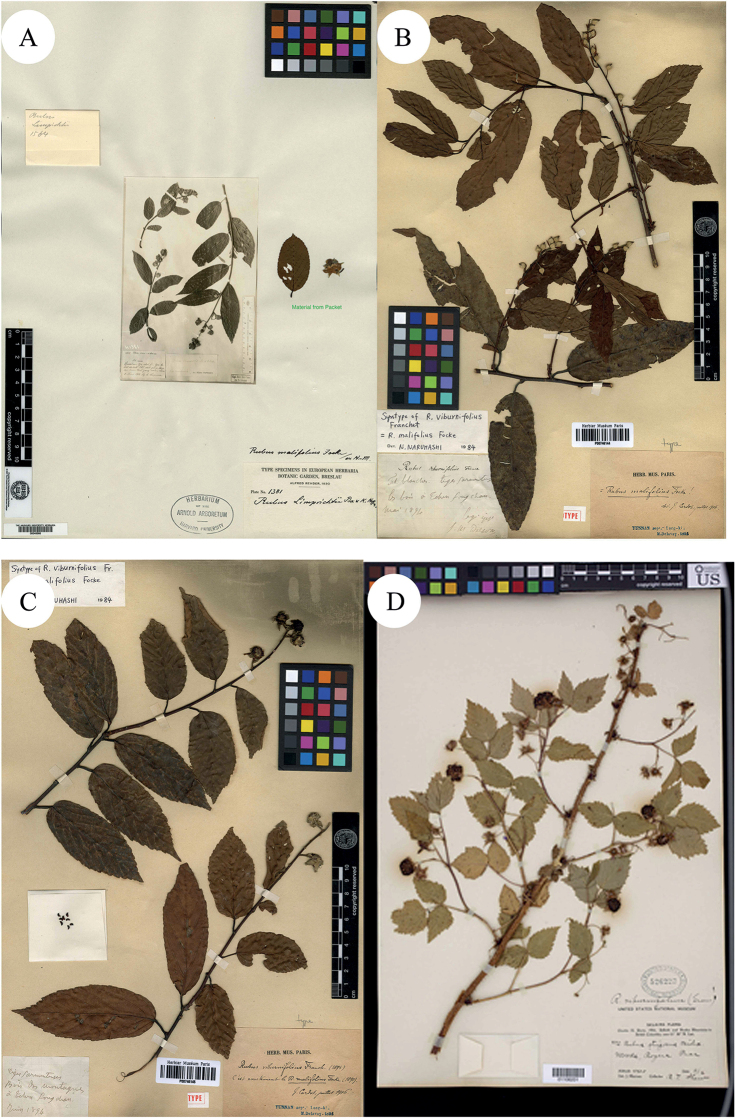
**A** Syntype of *R.limprichtii* Pax & K. Hoffm. **B–C** Syntypes of *R.viburnifolius* Franch. **D** Holotype of *B.viburnifolia* Greene. Specimen barcodes: A00040666, P00746144, P00746145 and US01106201, respectively.

Léveillé and Vaniot (1904) described *R.arbor* H. Lév. & Vaniot, based on the collection from Kouy-Tchéou Siao-tchang, Pin-fa, China, J. Cavaleri, May 1903, no. 1003 (Holotype E00010623!; Isotypes A00040529 (fragment with image of E00010623)!; E00313554!; K000737664!; Fig. 2A–D). Pax and Hoffmann (1922) described *R.limprichtii* Pax & K. Hoffm., based on the collection from Yatschou fu, Taldes Ya ho oberhalb Tschu schi ping, Hänge des Passes Tsiu gang schan, China, H.W. Limpricht, Jun 1914, no. 1564 (Syntype A00040666!; Fig. 3A). These two species were treated as synonyms of *R.malifolius* by Lu and Boufford (2003: 274) in Flora of China.

**Figure 4.**
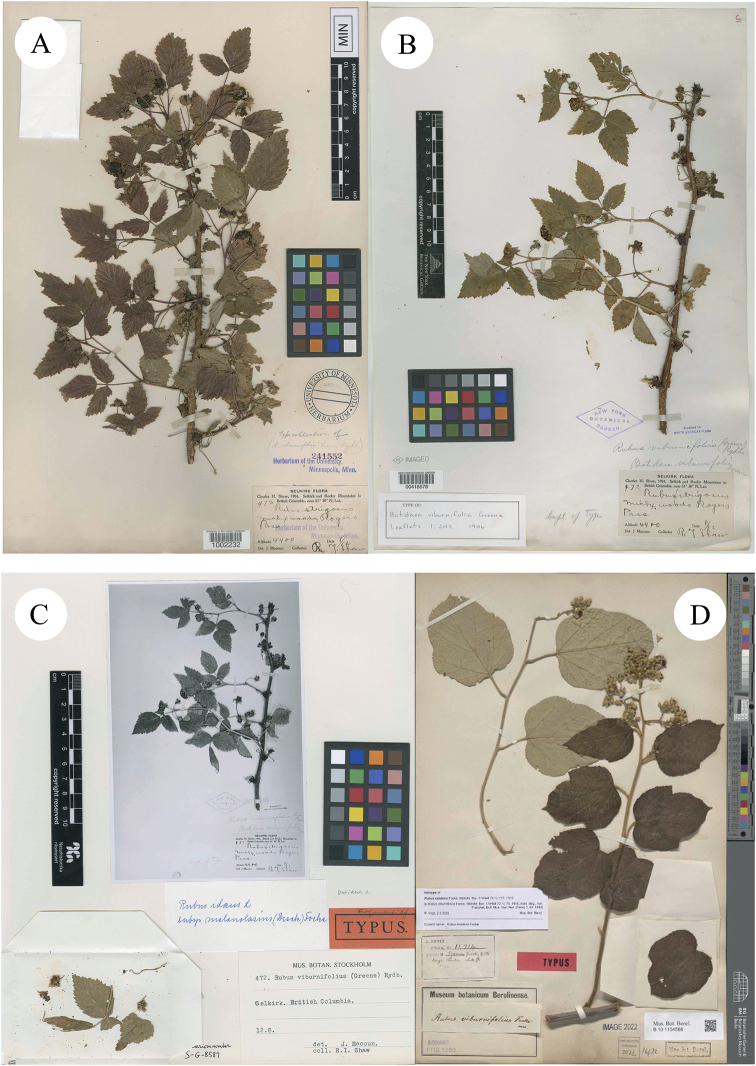
**A–C** Isotypes of *B.* v*iburnifolia* Greene **D** Holotype of *R.viburnifolius* Focke. Specimen barcodes: MIN1002232, NY00418578, S-G-8589 and B101154586, respectively.

**Figure 5.**
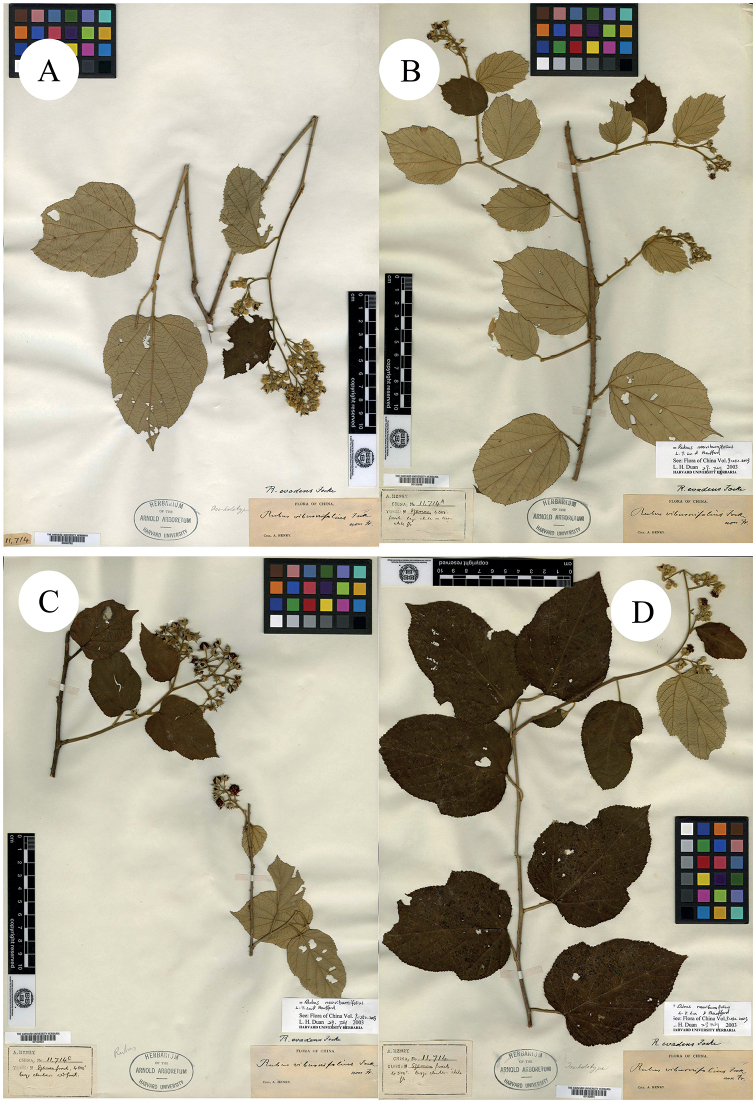
**A–D** Isotypes of *R.viburnifolius* Focke. Specimen barcodes: A00040762, A00132848, A00132850 and A00132854, respectively.

**Figure 6.**
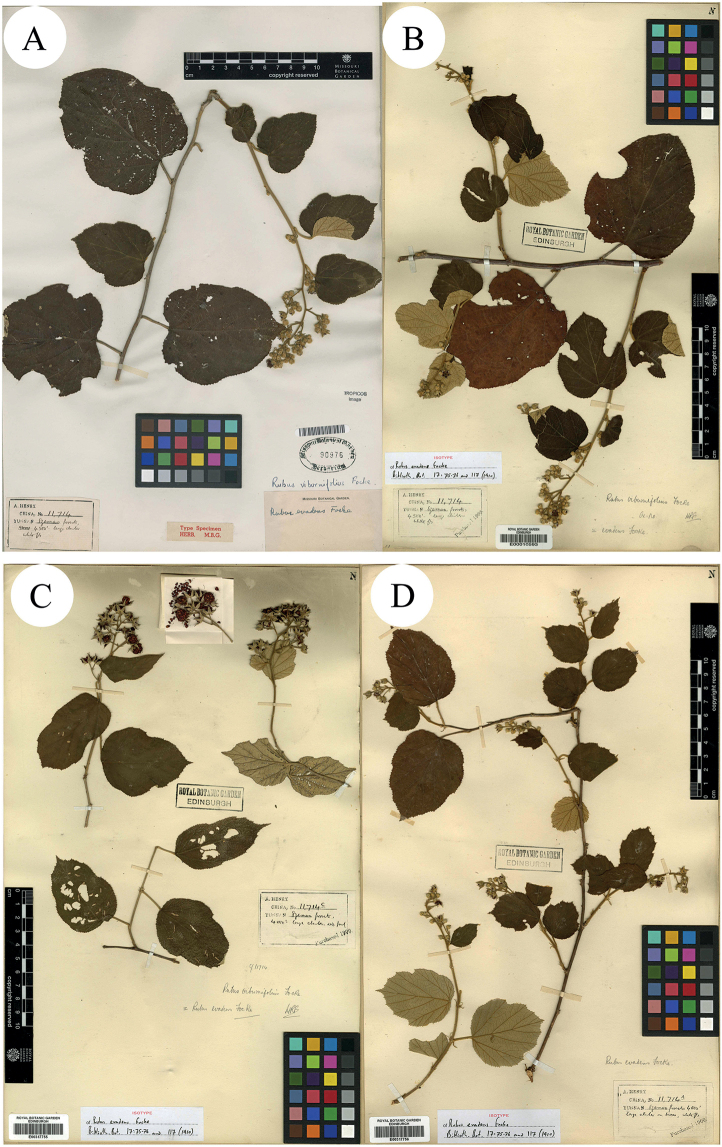
**A** Isotype of *R.viburnifolius* Focke **B–D** Isotypes of *R.evadens* Focke. Specimen barcodes: MO255250, E00010593, E00317755 and E00317756, respectively.

**Figure 7.**
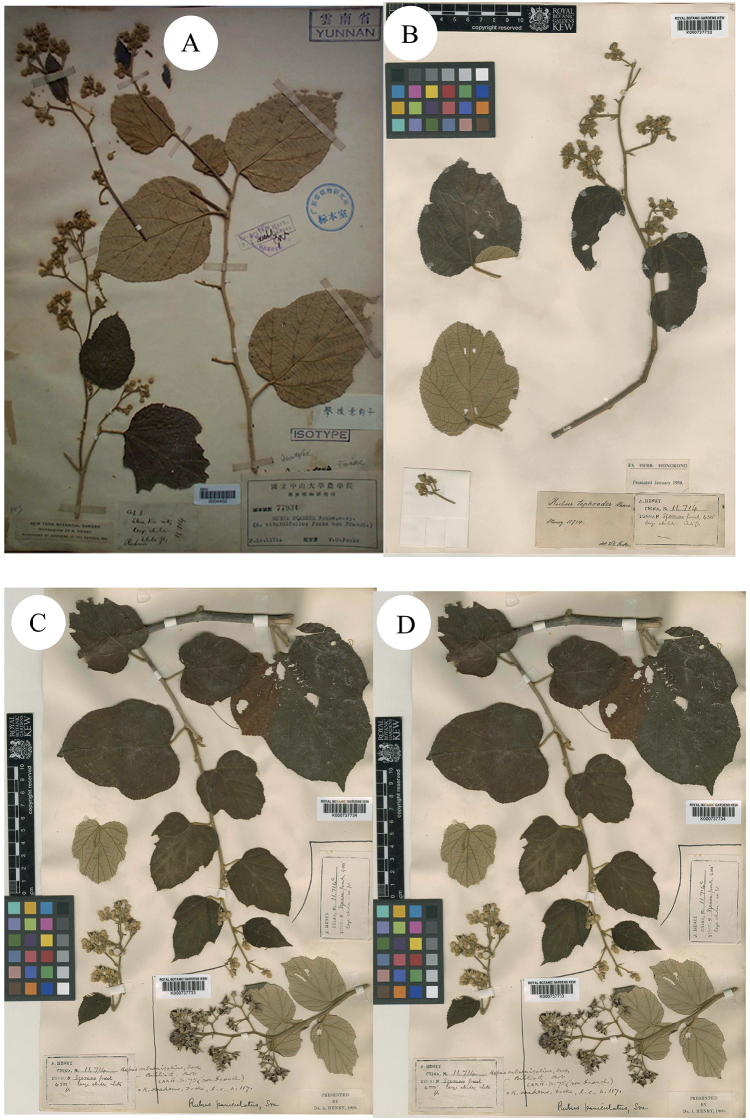
**A–C** Isotypes of *R.evadens* Focke. D Isotype of *R.viburnifolius* Focke. Specimen barcodes: IBSC0004402, K000737732, K000737733 (lower part of **C**), K000737734 (top part of **C**) and PE00020807, respectively.

**Figure 8.**
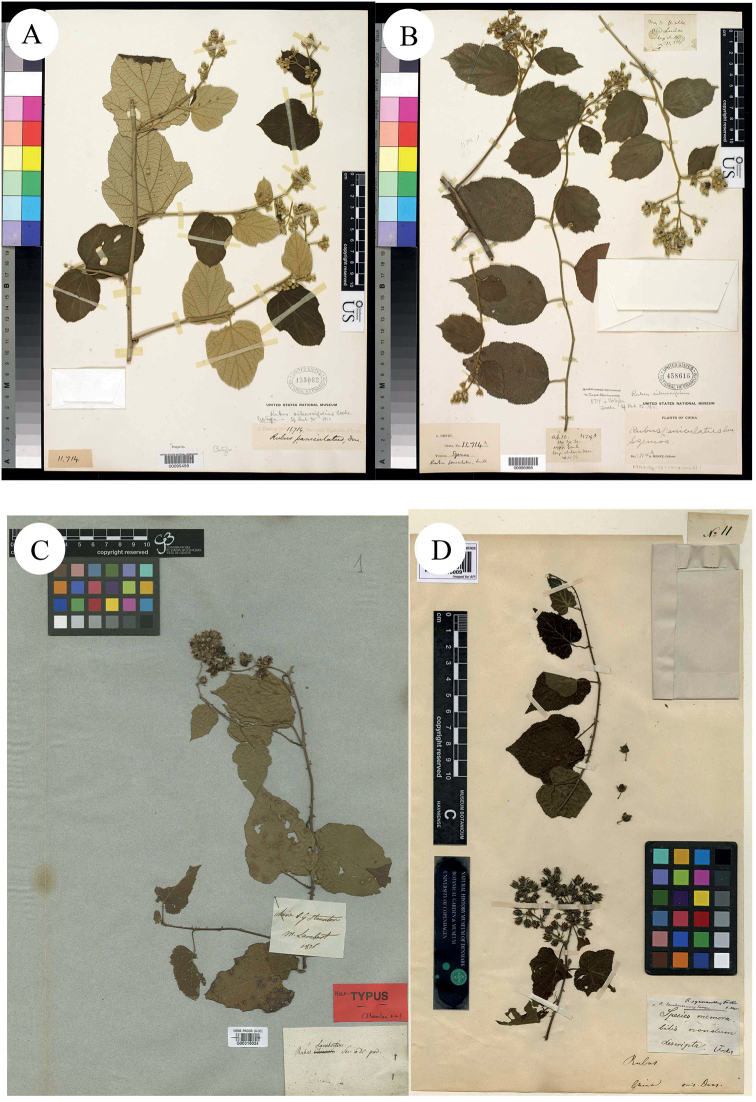
**A–B** Isotypes of *R.evadens* Focke; **C** Holotype of *R.lambertianus* Ser. **D** Holotype of *R.pycnanthus* Focke. Specimen barcodes: US00095499, US00996968, G00316024 and C10018009, respectively.

Franchet (1895) described *R.viburnifolius* Franch. in Bull. Mus. Hist. Nat. (Paris), based on the collection from les bois à Tchen-fong-chan. Yunnan, China, J.M. Delavay, Sep 1894, s. n. (Syntypes P00746144!, P00746145!; Fig. 3B–C). Greene (1906: 242) described *Batideaviburnifolia* Greene, based on the collection from Selkirk Mountains, US, C.H. Shaw, Aug 1904, no. 472 (Holotype US01106201!; Isotypes MIN1002232!; NY00418578!; S-G-8589 (fragment with image of NY00418578)!; Figs 3D and 4A–C). Then P.A. Rydberg (1913) merged the species into *Rubus* and proposed a new combination, *R.viburnifolius* (Greene) Rydb. However, it is a later homonym of *R.viburnifolius* Franch. and is, therefore, illegitimate under Art. 53.1 (Turland et al. 2018). In view of this, Berger (1925) treated it as a variety of *R.idaeus* L., which was named as R.idaeusvar.viburnifolius (Greene) A.Berger. Focke (1910) described *R.viburnifolius* Focke, based on the collection from Szemao, Yunnan, China, A. Henry, no. 11714, 11714A & B and 11714C (Holotype B101154586!; Isotypes A00040762!, A00132848!, A00132850!, A00132854!; MO-255250!; Figs 4D, 5A–D and 6A). Later, Focke rejected this *R.viburnifolius* (1910: 117; non-Franchet 1895, non-Rydberg 1913) and replaced it with *R.evadens* Focke (Isotypes E00010593!, E00317755!, E00317756!; IBSC0004402!; K000737732!, K000737733!, K000737734!; US00095499!, US00996968!; Figs 6B–D, 7A–D and 8A–B). Although *R.viburnifolius* Franch. is the legitimate name amongst these three names, the identification of their taxonomic status is still necessary.

*Rubuslambertianus* Ser. (1825) was published, based on the collection from China, Staunton, G. L., s. n. (Holotype G00316024!; Fig. 8C). Critical characteristics of the species were described as “Branchlets terete, thinly pubescent or subglabrous, with sparse, curved minute prickles. Leaves simple, cordate, base cordate, margin distinctly 3–5 lobed or undulate, serrulate. Stipules caducous, free. Inflorescences terminal usually cymose panicles, rachis and pedicels thinly pubescent, subglabrous, or glabrous. Calyx abaxially thinly pubescent, sepals ovate-lanceolate or triangular-lanceolate, margin entire, apex acuminate, margin of inner sepals grey tomentose. Petals white, obovate, glabrous, slightly shorter than or nearly as long as sepals, base clawed. Stamens many, somewhat shorter than petals; filaments broad, complanate. Pistils slightly shorter than or ca. as long as stamens, glabrous. Aggregate fruit red at maturity, subglobose, glabrous, with many drupelets, pyrenes small, prominently rugose”.

**Figure 9.**
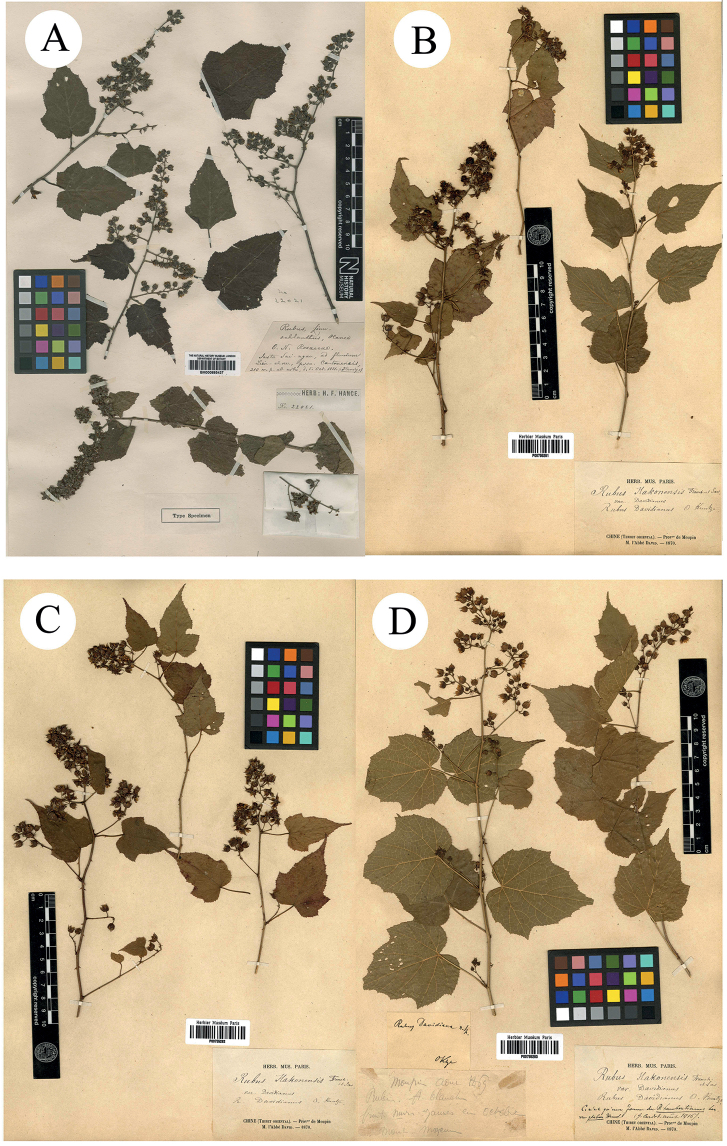
**A** Holotype of *R.ochlanthus* Hance **C–D** Syntypes of *R.davidianus* Kuntze. Specimen barcodes: BM000885437, P00755281, P00755282 and P00755283, respectively.

Hance (1882) described *R.ochlanthus* Hance, based on the collection from ad pagum Sai-ngau, secus fl. Lien-chau, Cantonensis, China, B.C. Henry, Oct 1881, no. 22021 (Holotype BM000885437!; Fig. 8D). According to the protologue, it was closely allied to *R.paniculatus* Sm., but was entirely distinct by the want of coloured indumentum, the much denser and more copious-flowered inflorescence and the smaller flowers. Focke (1874) described *R.pycnanthus* Focke, based on the collection from China, Duus, no.11 (Holotype C10018009!; Fig. 9A). In the protologue, Focke stated that *R.lambertianus* was different from *R.pycnanthus* by its lanceolate-acuminate sepals. However, these two species were treated as synonyms of *R.lambertianus* by Lu and Boufford (2003) in Flora of China.

Kuntze (1879) described *R.davidianus* Kuntze, based on the collection from Moupin, Su-Tchuen, China, A. David, Aug 1869, s. n. (Syntypes P00755281!, P00755282!, P00755283!; Fig. 9B–D). In Flora of China, Lu and Boufford (2003) considered it as a synonym of *R.crataegifolius* Bunge. However, its characters of terminal cymose panicles or axillary subracemes are different from those of *R.crataegifolius*. Thus, the taxonomic status of *R.davidianus* needs further research and its taxonomic treatment remains ambiguous.

## ﻿Materials and methods

We critically examined herbarium specimens of each species above, including all kinds of type specimens in A, BM, C, E, G, GH, IBS, K, MIN, MO, NY, P and US and checked them with protologues of each species.

## ﻿Results

The examination of herbarium specimens, identified as *R.arbor*, *R.limprichtii* and *R.malifolius*, indicated that they represented one species. According to Art. 11.4 of the “International Code of Nomenclature for Algae, Fungi and Plants (Shenzhen Code)” (Turland et al. 2018), *R.malifolius* Focke is the correct name of this species. In the same way, both *R.viburnifolius* (Greene) Rydb. and *R.viburnifolius* Focke are two later homonyms of *R.viburnifolius* Franch. and are, therefore, illegitimate under Art. 53.1 (Turland et al. 2018). Amongst these names with epithets such as “*viburnifolius*” and *R.viburnifolius* (Greene) Rydb., once described as *Batideaviburnifolia* Greene, this is characterised by “Leaves imparipinnate, 3–5-foliolate, terminal leaflet prominently petiolulate, petioles bristly, glandular-hispid and puberulent; abaxial surface of leaflets densely tomentose; stipules and bracts linear; terminal inflorescences short racemes, rarely several flower clusters in leaf axils; abaxial surface of calyx ± with needle-like prickles; pedicels densely glandular-hispid and somewhat bristly; petals white; fruit hemispherical, broad, red or yellowish, drupelets very numerous, comparatively small, falling together from the dry receptacle, pubescent”. These characters indicate that it is very closely related to *R.idaeus* and the differences are that the petiole, pedicel and abaxial surface of the calyx of *R.idaeus* have no glandular hairs. Thus, A. Berger (1925: 51) proposed R.idaeusvar.viburnifolius (Greene) A.Berger. as its correct name. On the other hand, Focke (1910: 75) proposed the name *R.viburnifolius* Focke to represent one Chinese *Rubus* species, but later he noticed his error and replaced it with *R.evadens*Focke (1910: 117). As the specimens of *R.viburnifolius* Franch. which is described from China, were unable to be viewed, this was treated as a suspicious species in Flora of China (Lu and Boufford 2003). We identified two specimens of *R.viburnifolius* Franch. in P and describe the characters of them as: “leaves simple, elliptic or oblong-elliptic, coarsely sharply serrate, base subrounded; stipules caducous, linear-oblong to ovate-lanceolate; terminal inflorescences racemes; rachis and pedicel densely tomentose-villous, gradually glabrescent, finally glabrous; bracts caducous, linear-oblong, apex acute to shortly acuminate; calyx abaxially densely tomentose-villous, sepals entire”. All of these characters indicate that *R.viburnifolius* Franch. is conspecific with *R.malifolius* and, therefore, *R.viburnifolius* Franch. is a later synonym of *R.malifolius*.

The examination of herbarium specimens, identified as *R.lambertianus*, *R.ochlanthus* and *R.pycnanthus* Focke, indicated that they represent the same species and, therefore, *R.lambertianus* is the correct name of this species. *R.davidianus* is a Chinese *Rubus* species described by Kuntze and three specimens of it have been identified in P, characters of them being described as: “shrubs; leaves simple, broadly ovate, rarely oblong-ovate, abaxially pilose, more densely so along veins, rarely glabrous, with sparse, minute prickles along mid-vein, adaxially pilose or hairy only along veins, cordate at base, margin distinctly 3–5-lobed or undulate, serrulate, apex acuminate; stipules and bracts narrower, less than 2 × 1 cm, linearly lobed; terminal inflorescences cymose panicles, axillary ones often subracemes, shorter, sometimes flowers few in clusters in leaf axils; pedicel 0.5–1 cm long; calyx abaxially thinly pubescent, sepals ovate-lanceolate or triangular-lanceolate, undivided; petals obovate, glabrous, slightly shorter than or nearly as long as sepals”. These characteristics are consistent with those of *R.lambertianus*, which indicate that *R.davidianus* is a later synonym of *R.lambertianus*, not *R.crataegifolius*.

In the process of *R.pycnanthus* Focke identification, we found that another plant, occurring in Haute-Vienne, Saint-Sulpice-les-Feuilles, Thias, Lamy, Angers, Maine-et-Loire, France, was also named as *R.pycnanthus* Genev. (Genevier 1880). Actually, Genevier (1868) firstly published it as *R.pyramidatus* Genev. Then he rejected it because Müller (1859) had published a name with the same epithet “*pyramidatus*” for a German plant. According to Art. 53.1 (Turland et al. 2018), *R.pycnanthus* Genev. is also a later homonym of *R.pycnanthus* Focke and, therefore, a new name, *R.loirensis* Ti R. Huang nom. nov., is proposed.

### ﻿Taxonomic treatment

#### ﻿1. Rubus lambertianus

Taxon classificationPlantaeRosalesRosaceae

Ser. Prodr. [A. P. de Candolle] 2: 567. 1825.

1A8D1A29-B5B2-5EF2-95D4-A711D347C0BE

R. davidianus Kuntze Meth. Sp.-Beschr. Rubus 58. 1879. syn. nov. Type: China, Moupin, Su-Tchuen, A. David, Aug 1869, s. n. (lectotype designated here by Ti R. Huang: P [P00755283]!; isolectotypes: P [P00755281, P00755282]!).
R. ochlanthus Hance J. Bot. 20: 260. 1882. Type: China, ad pagum Sai-ngau, secus fl. Lien-chau, Cantonensis, B.C. Henry, Oct 1881, no. 22021 (holotype: BM000885437]!).
R. pycnanthus Focke Abh. Naturwiss. Vereins Bremen 4: 196. 1874. non Genevier (1880: 210). Type: China, Duus, no.11 (holotype: C [C10018009]!).

##### Type.

China, Staunton, G. L., s. n. (holotype: G [G00316024]!).

##### Distribution and habitat.

*Rubuslambertianus* grows in slopes, roadsides, montane valleys, grasslands, thickets and forest margins. Its elevation ranges from low to medium. In China, it is distributed in Anhui, Fujian, Guangdong, Guangxi, Guizhou, Hainan, Henan, Hubei, Hunan, Jiangsu, Jiangxi, Taiwan, Yunnan and Zhejiang Provinces and overseas in Japan.

##### Phenology.

Flowering from July to August and fruiting from September to November.

##### Taxonomic notes.

*Rubuslambertianus* is similar to *R.laxus* Focke, the differences being: the latter has leaves narrowly ovate; pedicel 1–2 cm long; sepals ovate or ovate-triangular, outer sepals pinnately laciniate, petals slightly pubescent.

##### Additional specimens examined.

**China. Sichuan.** 1934, T.H.Tu, no. 1604 (IBSC0324688); 15 October 1935, Xianyu, no. 6908 (NAS00366117);15 October 1935, Xianyu He, no. 6908 (NAS00366117); 20 August 1963, Chuanxi Expedition Kechien Kuan Wentsai Wang et al., no. 2437 (PE02092824); 12 June 2014, Shuren Zhang et al., no. 1833 (PE01918855); 22 September 1978, Ya’an Expendition, s.n. (SM707005133, SM707005134). **Yunnan.** Shen’e Liu, no. 14014 (IBSC0324680); 25 June 1946, Shen’e Liu, no. 15383 (IBSC0324683); 8 August 1938, Tetsun Yu, no. 17291 (KUN711083); 28 July 1985, Zhanhe Ji, no. 306 (PE01828470); 24 May 1998, TianGang Gao, no. 1681 (PE01828469); 25 August 2002, Hong Wang, no. 6120 (PE01813595).

#### ﻿2. Rubus malifolius

Taxon classificationPlantaeRosalesRosaceae

Focke Hooker’s Icon. Pl. 20: t. 1947. 1890.

1FCF8744-6A10-5AFB-B6BA-D597BB18CBF6

R. viburnifolius Franch. Bull. Mus. Hist. Nat. (Paris) 1: 63. 1895. Non Focke (1910: 75) nec Rydberg (1913: 446) Type: China, les bois à Tchen-fong-chan. Yunnan, J.M. Delavay, Sep 1894, s. n. (lectotype P00746144! (designated here by Ti R. Huang); isolectotype P00746145!).
R. arbor H. Lév. & Vaniot Bull. Soc. Bot. France 51: 217. 1904. Type: China, Kouy-Tchéou Siao-tchang, Pin-fa, J. Cavaleri, May. 1903, no. 1003 (holotype: E [E00010623]!; isotypes: A [A00040529] (with an image of E00010623)!, E [E00313554]!, K [K000737664]!).
R. limprichtii Pax & K. Hoffm. Repert. Spec. Nov. Regni Veg. Beih. 12: 406. 1922. Type: China, Yatschou fu, Taldes Ya ho oberhalb Tschu schi ping, Hänge des Passes Tsiu gang schan, H.W. Limprich, Jun. 1914, no. 1564 (holotype: A [A00040666]!).

##### Type.

China, Chienshih, Hubei, A. Henry, 1885, no. 5794 (lectotype designated here by Ti R. Huang: K [K000737665]!; isolectotypes: BM [BM000622260]!, GH [GH00040667]!, US [US00097945]!).

##### Distribution and habitat.

*Rubusmalifolius* grows in slopes, ravines, stream sides, montane valleys, forests and thickets. Its elevation ranges from 400–2200 m. It is endemic to China and is distributed in Guangdong, Guangxi, Guizhou, Hubei, Hunan, Sichuan and Yunnan Provinces.

##### Phenology.

Flowering from May to June and fruiting from July to August.

##### Taxonomic notes.

*Rubusmalifolius* is similar to *R.preptanthus* Focke, the differences being: the latter has leaves narrowly obovate or broadly ovate-lanceolate to narrowly lanceolate, base rounded to subtruncate; stamens glabrous or anthers slightly villous; styles ca. as long as or slightly longer than stamens.

##### Additional specimens examined.

**China. Yunnan.** E.E. Maire, no. 104 (IBSC0340297); 15 July 1934, H.T.Tsai, no. 62641 (IBSC0340298); 16 May 1973, Zhihao Hu, no. 1382 (IBSC0340299; PE01833218); 24 May 1973, Bixing Sun et al., no. 401 (IBSC0340301; PE01833217); 14 August 1934, H.T.Tsai, no. 62641 (NAS00366395); 8 May 1964, Wang Shouzheng, no. 205 (KUN711739); 4 June 1959, Anquan Wu, no. 8240 (KUN711742); 15 July 1934, H.T.Tsai, no. 62641 (KUN757822; PE01833216, PE00252217); 12 August 1947, K.M. Feng, no. 11103 (PE00252220); 13 April 1940, C.W.Wang, no. 88450 (PE00252221); 20 August 1985, Zhanhe Ji Shunyin Song & Xintang Ma, no. 601 (PE01833194, PE01833216); 6 April 1993, Yumin Shui, no. 2131 (PE01840835). **Sichuan**: 1932, T.T. Yu, no. 848 (IBSC0340264; PE00252196); 12 May 1941, Wenpei Fang, no. 16617 (IBSC0340273; PE00252199); 12 May 1941, Wenpei Fang, no. 16619 (IBSC0340277; PE00252200); Jinguiyuan, Huangjing, Gulin County, 29 May 2010, PE-GulinExpediton Team, no.40 (PE01864955); Xixi, Shuiwei, Xuyong County, Liang Zhang Xinmao Zhou & Wenbin Ju, no. HGX14303 (CDBI0226242; CDBI0226243). **Guizhou**: 22 June 1935, S.W.Teng, no. 640 (IBSC0340289); 3 July 1936, S.W.Teng, no. 90506 (IBK00065627, IBK00065634; IBSC0340283; NAS00366394; KUN711716; PE00252176); 14 July 1931, S.S.Sin, no. 51134 (IBSC0340287; IBSC0340291); 22 May 1930, Y.Tsiang, no. 5030 (IBSC0340288); 13 June 2003, Ye He, no.1-197 (PE01833201); 29 May 2016, Xinyun Lu, no. KKS1602173 (ZY0000066).

#### ﻿3. Rubus loirensis

Taxon classificationPlantaeRosalesRosaceae

T.Huang nom. nov.

96BC2FC3-FAB7-5F51-B068-DE80D45D832C

urn:lsid:ipni.org:names:77306480-1

##### Replaced synonym.

*R.pycnanthus* Genev. (1880: 210), non Focke (1874: 196).

##### Type.

France, Haute-Vienne, Saint-Sulpice-les-Feuilles, Thias, Lamy, Angers, Maine-et-Loire.

##### Distribution and habitat.

*Rubusloirensis* grows in woods, hedges, shale and granite. In France, it is distributed in Haute-Vienn, Maine-et-Loire and Loire-Inférieure.

##### Phenology.

Flowering from June to July.

##### Taxonomic notes.

L.G. Genevier (1868: 192) wrongly reported this species as *R.pyramidatus* P.J. Müll. in the Mém. Soc. Acad. Maine Loire. Later, L.G. Genevier corrected the error and proposed a replacement name *R.pycnanthus*.

*R.loirensis* is similar to *R.anadenes* P.J.Müll. ex Genev., the differences being: the former has petals wider, the stamens exceeding the styles and erect peduncles. It is also similar to *R.atrocaulis* P.J.Müll., the differences being: the former petals white and it is different from *R.stereacanthos* P.J.Müll. ex Genev. by its narrow panicles.

#### ﻿4. Rubus evadens

Taxon classificationPlantaeRosalesRosaceae

Focke, Biblioth. Bot. 17 (Heft 72 part I): 117 (75–76; fig. 27). 1910).

42CB0B2D-153E-5DC5-B564-A81B6FD9E9F1

R. nanopetalus Cardot, Notul. Syst. (Paris) 3: 300. 1917. Type: China, Lao-tsou-te-outze, Yunnan, Bons d’Anty, s. n. (holotype: P [P00746126]!).
R. viburnifolius Focke var. apetalus Y. Gu & W.L. Li, Bull. Bot. Res., Harbin 20(2): 122. 2000. Type: China, Yuanyang County, Yunnan, 1996, Yin Gu et al., no. 018 (holotype: NAS (JSBI); Jing dong, 1996, Yin Gu et al., no. 240, 241 (paratypes: NAS (JSBI)); Yuanyang-Lǜchun divide (元阳绿春分水岭), 1996, Yin Gu et al., no. 030, 033 (paratypes: NAS (JSBI)).

##### Replaced synonym.

*R.viburnifolius*Focke (1910: 75), non Franchet (1895: 63) nec Rydberg (1913: 446). — *Rubusneoviburnifolius* Lu & Boufford (2003: 252).

##### Type.

China, Szemao, Yunnan, A. Henry, no. 11714, 11714A & B and 11714C (holotype: B [B101154586]!; isotypes: A [A00040762, A00132848, A00132850, A00132854]!, E [E00010593, E00317755, E00317756]!, IBSC [IBSC0004402]!, K [K000737732, K000737733, K000737734]!, MO [MO-255250]!, NY [NY00429679]!, PE [PE00020807]!, SYS [SYS00076267]!, US [US00996968, US00095499]!).

##### Distribution and habitat.

*Rubusevadens* grows in dry slopes and mixed forests. Its elevation ranges from 1200 to 3000 m. It is endemic to southern Yunnan.

##### Phenology.

Flowering from June to July and fruiting from August to October.

##### Taxonomic notes.

*Rubusevadens* is similar to *R.paniculatus* Smith, the differences being: the latter has leaves ovate to narrowly ovate, apically acuminate; petioles 2–4 cm long; flowers to 18 mm in diam.; terminal cymose panicles broad, lax.

#### ﻿5. Rubus idaeus L. var. viburnifolius

Taxon classificationPlantaeRosalesRosaceae

(Greene) Greene ex A. Berger, New York Agric. Exp. Sta. Bull. 2: 51. 1925.

095F58B4-99F3-5B99-A68B-5FB2EE884957

R. viburnifolius (Greene) Rydb. (1913: 446) ≡ BatideaviburnifoliaGreene (1906: 242) Type: US, Selkirk Mountains, C.H. Shaw, Aug 1904, no. 472 (holotype: US [US01106201]!; isotypes: MIN [MIN1002232]!, NY [NY00418578]!, S [S-G-8589]! (with image of NY00418578).

##### Distribution and habitat.

Rubusideausvar.viburnifolius (Greene) Greene ex A.Berger grows in woods. It is distributed in western North America, Alaska to Mackenzie, Montana and south to British Columbia and perhaps to Wyoming and Utah.

##### Phenology.

Unknown.

##### Taxonomic notes.

Rubusideausvar.viburnifolius (Greene) Greene ex A.Berger is treated as a variety of *R.ideaus* and is similar to R.ideausvar.peramoenus (Greene ex Fedde) Fernald. The differences are: canes glabrous or puberulent and more or less densely bristly; leaflets also green on both sides or somewhat tomentose underneath when young, but strongly veined beneath and more or less plicate; the former has inflorescence rachis and pedicels with glandular hairs; abaxial surface of calyx without glandular hairs; branchlets, petioles and pedicel with sparse prickles or nearly unarmed.

## ﻿Discussion

Lu and Boufford (2003: 285) listed *R.viburnifolius* Franch. at the end of Flora of China to indicate that it had been described from Yunnan, China, but they have not seen any specimens and are, therefore, unable to treat it. Meanwhile, they stated that further revision of this species was necessary. In this paper, we carried out critical examinations of herbarium specimens, from which morphological characters of *R.arbor*, *R.limprichtii*, *R.malifolius* and *R.viburnifolius* were studied. Morphological characters of *R.arbor*, *R.limprichtii*, *R.malifolius* and *R.viburnifolius* Franch. indicate that they represent the same species and, therefore, *R.malifolius* is the correct name according to the “International Code of Nomenclature for Algae, Fungi and Plants (Shenzhen Code)” (Turland et al. 2018). Additionally, both *R.viburnifolius* (Greene) Rydb. and *R.viburnifolius* Focke are later homonyms of *R.viburnifolius* Franch., in which *R.viburnifolius* (Greene) Rydb. was replaced by R.idaeusvar.viburnifolius and *R.viburnifolius* Focke was replaced by *R.evadens*.

*Rubusdavidianus* was treated as a synonym of *R.crataegifolius* Bunge by Lu and Boufford (2003: 236) in Flora of China. However, examination of herbarium specimens indicates that there are distinct differences between the two species. The differences are: the former has inflorescences with terminal cymose panicles, axillary ones often subracemes, shorter, sometimes flowers few in clusters in leaf axils; stipules and bracts narrower, less than 2 × 1 cm, linearly lobed; the latter has inflorescences terminal, rarely axillary, short racemes or flowers several in cluster; stipules and bracts linear, entire. Thus, we conclude that *R.davidianus* and *R.crataegifolius* should represent two different species of *Rubus* and *R.davidianus* should be a synonym of *R.lambertianus*. Three specimens stored under *R.pyramidatus* P.J. Müll. in P were found; however, the specimens stored under either *R.pycnanthus* Genev. or *R.pyramidatus* Genev. could not be traced. Based on the existing characters of *R.pyramidatus* P.J. Müll. and *R.pycnanthus* Genev., we can identify that these two species are different from that of *R.pycnanthus* Focke. Though there are old attempts to synonymise *R.pycnanthus* Genev., the taxonomic status of *R.pycnanthus* Genev. should still be studied.

Species identification of *Rubus* species indicates that many homonyms and synonyms still exist in the genus *Rubus*, especially when they were more common in the 18^th^, 19^th^ and 20^th^ century. This could be interpreted in three ways. First, because of the propensity for interspecific hybridisation, polyploidy and apomixis, morphological characters of the species under this genus are highly variable and diverse. This makes species division and identification very difficult. Second, the original publications of species are often kept in the libraries of various scientific research institutions and some original publications are even kept in private collections. Objectively, this increases the difficulty for people to obtain and read the information of species publications. Third, examination of type specimens could not be easily accessed since digitisation of specimens was not yet widespread. Therefore, species names of *Rubus*, once not given sufficient attention or had not been discovered, should be emphasised in further taxonomic studies, using the integrative morphological characters and integrative systematics.

## Supplementary Material

XML Treatment for Rubus lambertianus

XML Treatment for Rubus malifolius

XML Treatment for Rubus loirensis

XML Treatment for Rubus evadens

XML Treatment for Rubus idaeus L. var. viburnifolius
